# Orthodontic Management of Midline Diastema in Mixed Dentition

**DOI:** 10.5005/jp-journals-10005-1083

**Published:** 2011-04-15

**Authors:** Arvind Kumar, Raghvendra M Shetty, Uma Dixit, K Mallikarjun, Anil Kohli

**Affiliations:** 1 Reader, Department of Pedodontics and Preventive Dentistry, Rama Dental College Hospital and Research Center, Kanpur, Uttar Pradesh, India; 2 Reader, Department of Pedodontics and Preventive Dentistry, Chhattisgarh Dental College and Research Institute, Rajnandgaon, Chhattisgarh, India; 3 Professor, Department of Pedodontics and Preventive Dentistry, Dr DY Patil Dental College and Hospital, Navi Mumbai, Maharashtra, India; 4 Professor, Department of Pedodontics and Preventive Dentistry, Rama Dental College, Hospital and Research Center, Kanpur, Uttar Pradesh, India; 5 Professor and Head, Department of Pedodontics and Preventive Dentistry, Rama Dental College, Hospital and Research Center, Kanpur, Uttar Pradesh, India

**Keywords:** Midline diastema, Mesiodens, Orthodontic treatment.

## Abstract

Midline diastema is a space between the central incisors. Although physiologic transitory maxillary midline diastema is observed in children during eruption of maxillary anterior teeth in most cases, it is self-corrected after eruption of maxillary canines. However, midline diastema unrelated to the eruption of teeth has been observed owing to various etiologic factors. Treatment to align the central incisors depends on the predisposing factors.

## INTRODUCTION

Midline diastema is a space between the central incisors. The prevalence of midline diastema is high in children and it decreases with age.^1^ Although physiologic transitory maxillary midline diastema is observed in children during eruption of maxillary anterior teeth in most cases, it is self-corrected after eruption of maxillary canines. However, midline diastema unrelated to the eruption of teeth has been observed owing to various etiologic factors, such as supernumerary teeth, congenital absence of permanent teeth, deleterious oral habits, high frenal attachment and others such as peg-shaped laterals and microdontia.^2^

Panoramic and periapical radiographs are necessary to evaluate patient’s dental age, unerupted supernumerary teeth, missing teeth or abnormal eruption path.^3^

Three case reports of midline diastema and their treatment plan are presented here.

## CASE REPORT

### Case 1

A 13-year-old male patient reported to our department with chief complaint of extra teeth in upper anterior region of mouth with spacing.

**Fig. 1:**
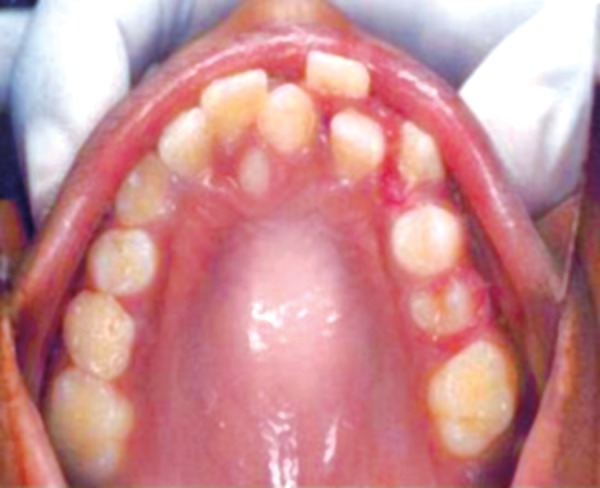
Intraoral examination showing two fully erupted mesiodens

**Fig. 2:**
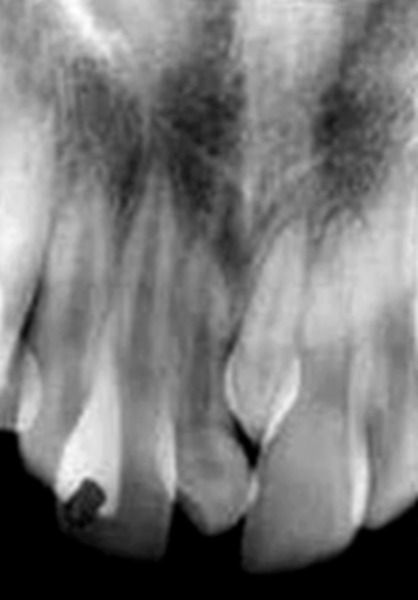
Periapical radiograph showing absence of unerupted supernumerary teeth

Intraoral examination revealed two fully erupted mesiodens, one behind another (Fig.1), leading to flared maxillary right central incisor and diastema of 4 to 5 mm. The patient had Angle’s class I molar relationship. Radiographic examination ruled out presence of any other unerupted supernumerary teeth (Fig. 2). It was diagnosed that mesiodens caused flaring of right maxillary central incisor. Hence, it was decided to intercept the clinical condition by extracting the mesiodens followed by space closure.

After consent was obtained from parents, the mesiodens were extracted under local anesthesia and patient was called after one month for further treatment.

**Fig. 3:**
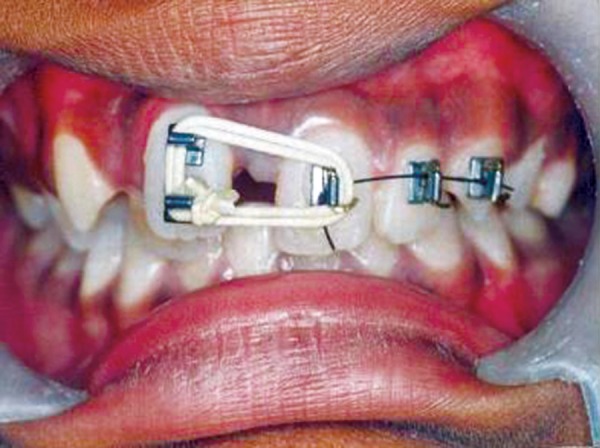
Elastic thread used to achieve mesial bodily movement of right central incisor

Upon arrival, patient underwent thorough oral prophylaxis and was taken up for space closure using simple fixed appliance therapy. The central incisors, left lateral incisor and canine were bonded with brackets. Elastic thread was used to achieve mesial bodily movement of right central incisor. Anchorage was taken on the left side to prevent bodily movement of left central incisor (Fig. 3).

**Fig. 4:**
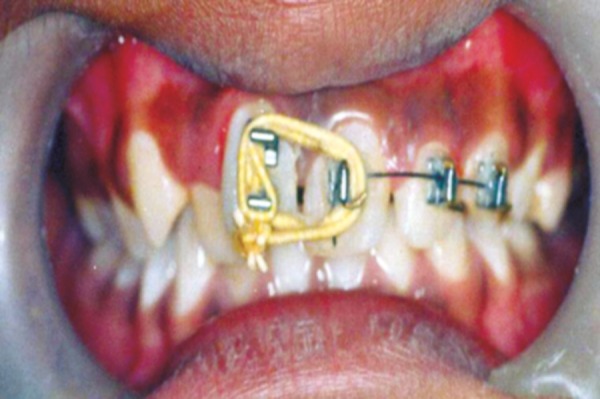
Patient followed up after 2 months

The patient was followed up every 15 days for first 2 months then monthly till 5 months (Fig. 4). During this period, as the space closed, brackets were removed and lingual side fixed retention appliance was given.

**Fig. 5:**
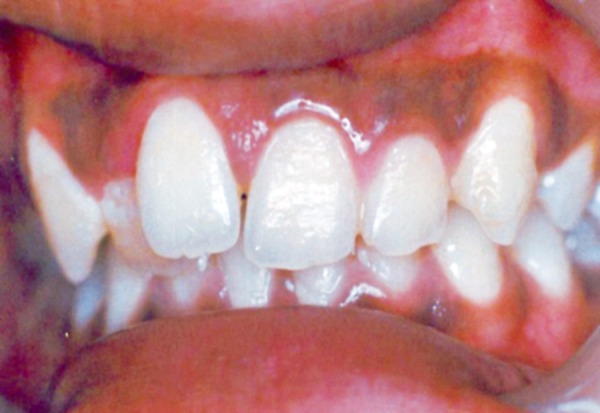
Post-treatment clinical appearance

**Fig. 6:**
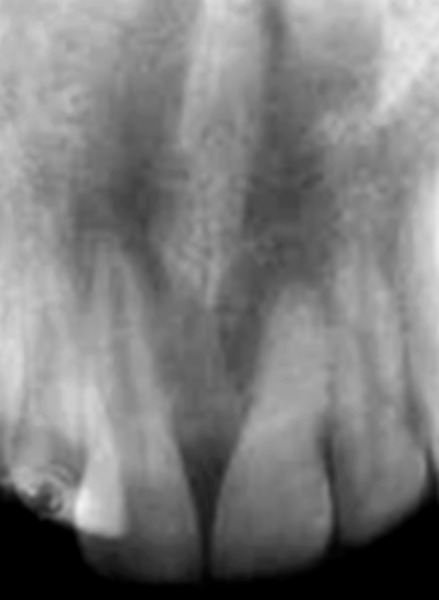
Post-treatment periapical radiograph

The patient was followed up for a period of 8 months, at the end of which remarkable improvement in esthetics was achieved (Figs 5 and 6).

### Case 2

**Fig. 7:**
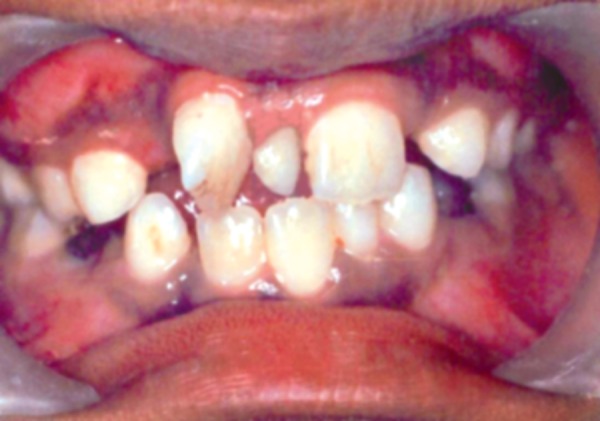
Intraoral examination showing mesiodens with 180° rotated central incisor

**Fig. 8:**
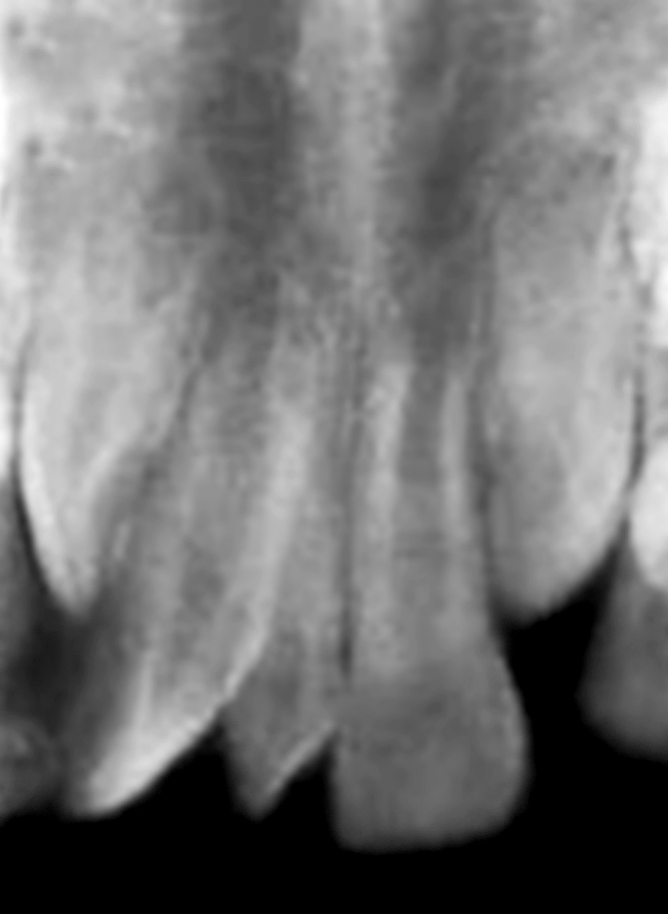
Periapical radiograph showing absence of any other supernumerary teeth

In second case, a 8-year-old male patient reported with chief complaint of extra and rotated teeth in upper anterior region of mouth. Intraoral examination revealed mesiodens with 180° rotated right maxillary central incisor (Fig. 7). The patient had Angle’s class I molar relationship. Radiographic examination ruled out presence of any other unerupted supernumerary teeth (Fig. 8). It was diagnosed that the mesiodens caused ectopic eruption of right central incisor; hence it was decided to intercept the clinical condition by extracting the mesiodens, followed by space closure with derotation of right central incisor.

After consent was obtained from the parents, the mesiodens was extracted under local anesthesia and patient was called after one month for further treatment.

Upon arrival the healing of extraction wound was satisfactory. Derotation of right central incisor was started with brackets attached on labial and lingual side of the rotated maxillary right central incisor, and on buccal side of first maxillary molar on both right and left side.

**Fig. 9:**
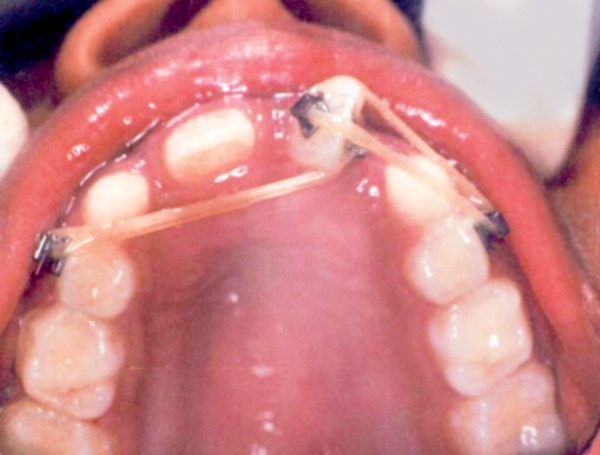
Elastic bands placed for derotation

Elastic bands were placed from labial side of the incisor to the right side of maxillary molar and lingual side of the central incisor to left side of maxillary molar. More elastic bands were placed on lingual side so as to exert more force and derotate the incisor. Right side elastic acted as an anchorage (Fig. 9).

Patient was provided with elastic bands and was asked to change elastics every week, and instructions regarding oral hygiene maintainance were given.

The patient was followed up every month for a period of 4 months. During this period maxillary right central incisor derotated.

**Fig. 10:**
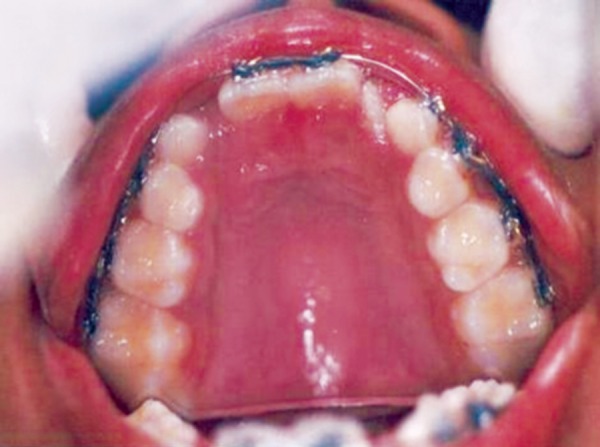
Full arch maxillary anchorage

Full arch maxillary anchorage was taken to avoid undesired rotation of derotated incisor, mesial migration of the molars and for proper arch alignment (Fig. 10).

**Fig. 11:**
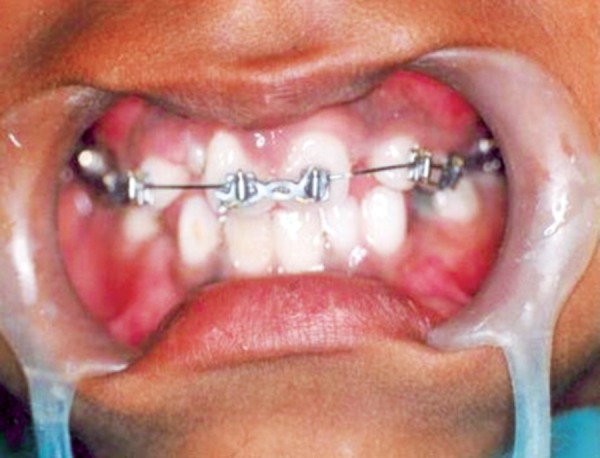
Post-treatment clinical appearance after 8 months

The patient was followed up for a total period of 8 months at the end of which remarkable improvement in esthetics was achieved (Fig. 11).

**Fig. 12:**
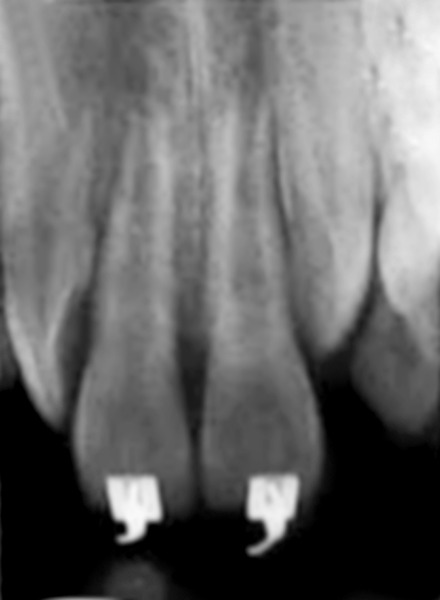
Periapical radiograph showing no bone resorption with proper alignment

Post-treatment radiograph shows proper alignment of root of central incisors without any bone resorption (Fig. 12).

### Case 3

**Fig. 13:**
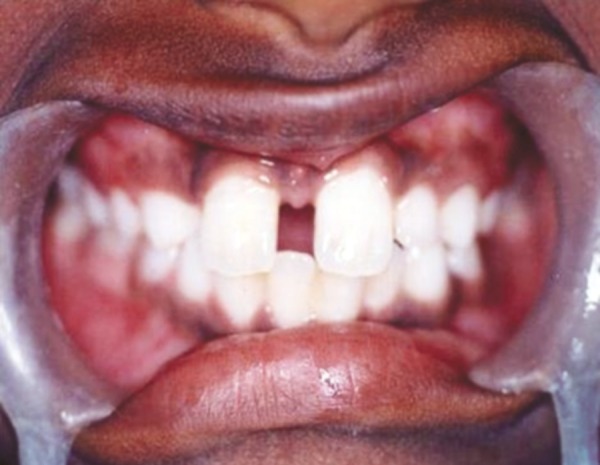
Intraoral examination showing midline diastema

**Fig. 14:**
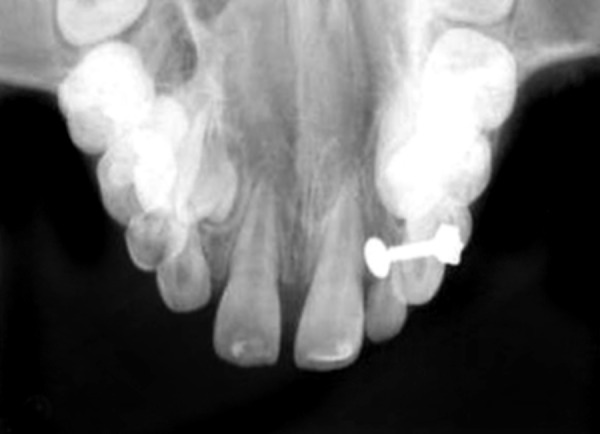
Occlusal radiograph showing bilateral congenital absence of lateral incisors

In another case, a 11-year-old female patient reported with chief complaint of space between upper anterior teeth. Intraoral examination revealed midline diastema of 3 to 4 mm. With over-retained maxillary left deciduous lateral incisor (Fig. 13). The patient had Angle’s class I molar relationship. The radiographic examination revealed congenital absence of permanent lateral incisors on both sides (Fig. 14).

It was diagnosed that congenital absence of lateral incisors were responsible for flaring of central incisors and midline diastema.

Patient underwent thorough oral prophylaxis and was taken up for diastema closure using simple fixed appliance therapy.

**Fig. 15:**
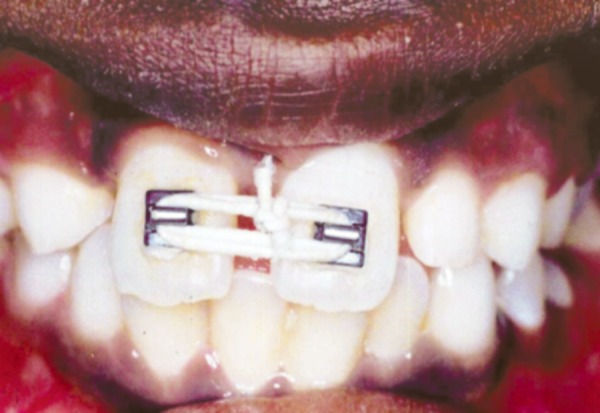
Brackets placed horizontally

The central incisors were bonded with Begg’s bracket on the labial surface. The brackets were horizontally placed, so that it can engage the elastic thread into it and prevent the thread from the slipping out and irritating the soft tissue (Fig. 15). The patient was recalled after one week and it was observed that patient had adapted well to the appliance.

The patient was followed up every 15 days for first 3 months then monthly till 6 months.

**Fig. 16:**
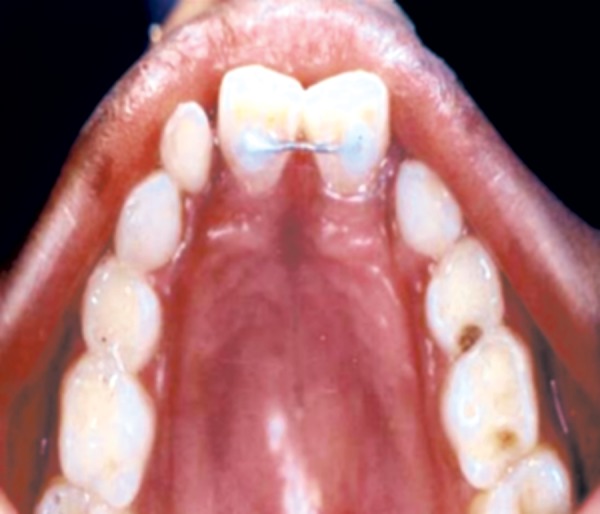
Lingual side fixed retention given

**Fig. 17:**
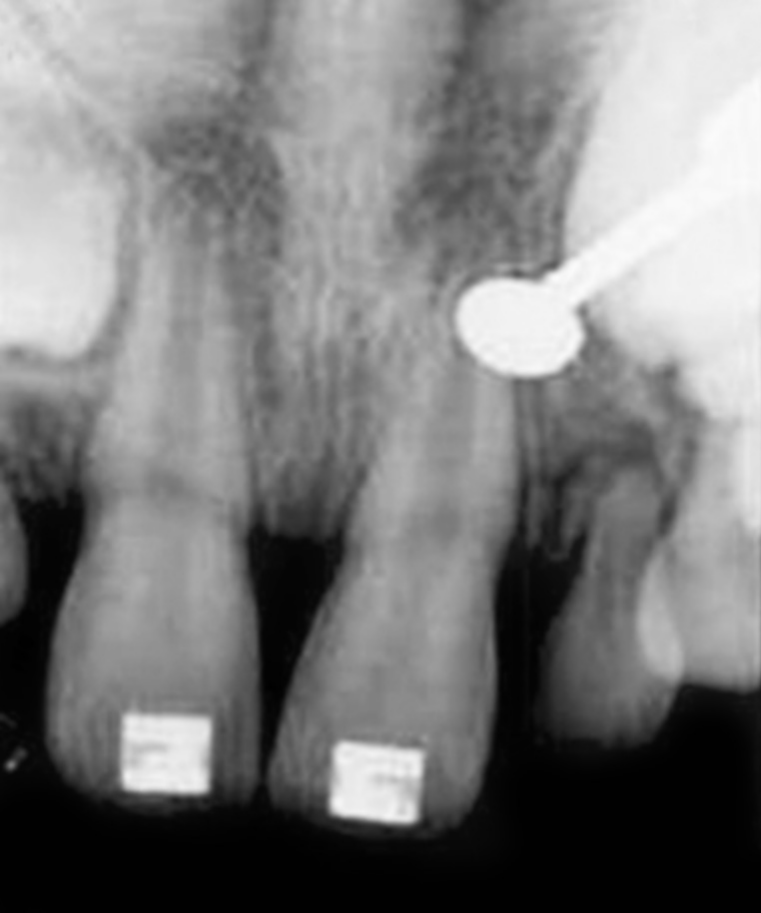
Post-treatment periapical radiograph showing closure of midline diastema

During this period, as the space closed, the brackets were removed and lingual side fixed retention was given (Figs 16 and 17). The patient was followed up till eruption of permanent canine.

## DISCUSSION

It has been reported that mesiodens may deflect the eruption pattern of maxillary central incisors or it may physically move the incisors laterally to create midline diastema.^4^

The congenital absence of teeth might also lead to the midline diastema.^2^

A number of treatment modalities for midline diastema correction have been reported in litreature.^5-7^

The ideal treatment of most of the diastema cases involves bodily approximation of the incisor with full bonded and bracketed orthodontic arch appliance. However, in certain cases sectional arch wire technique may be useful. Consideration should be given for alignment of midline.^8^

Since most diastema closure recurs after even the best managed treatment, permanent retention is required in most cases. Lingual bonded fixed retainer, straight section wire, ligature wire, etc. has been recommended for retention therapy.^9^

In present cases after diastema closure lingual bonded straight section wire was used for retention.

## CONCLUSION

With this successful completion of treatment of midline diastema in these three patients, we conclude that early-developing malocclusions should be intercepted with the goal of restoring normal occlusion. The timing and degree of interception are the major problems of interceptive stages, which if dealt properly, can produce positive result in the mixed dentition.
